# GABA_B_ receptor antagonist promotes hippocampal neurogenesis and facilitates cognitive function recovery following acute cerebral ischemia in mice

**DOI:** 10.1186/s13287-020-02059-x

**Published:** 2021-01-07

**Authors:** Dan Song, Yaohua Chen, Cheng Chen, Lili Chen, Oumei Cheng

**Affiliations:** 1 Department of Neurology, The First Affiliated Hospital of Chongqing Medical University, Chongqing, 400016 China; 2 Laboratory Research Center, The First Affiliated Hospital of Chongqing Medical University, Chongqing, 400016 China

**Keywords:** GABA_B_ receptor, Cerebral ischemia, Neurogenesis, Cognitive function, Neural stem cells

## Abstract

**Purpose and background:**

Previous studies have suggested that promoting endogenous neurogenesis has great significance for the recovery of cognitive dysfunction caused by cerebral ischemia (CI). Pharmacological inhibition of GABA_B_ receptor can enhance neurogenesis in adult healthy and depressed mice. In the study, we intended to investigate the effects of GABA_B_ receptor antagonists on cognitive function and hippocampal neurogenesis in mice following CI.

**Methods:**

Adult mice were subjected to bilateral common carotid artery occlusion (BCCAO) for 20 min to induce CI and treated with CGP52432 (antagonist of GABA_B_ receptor, CGP, 10 mg/kg intraperitoneal injection) starting 24 h after CI. The Morris water maze test was performed to test spatial learning and memory at day 28. Immunofluorescence was applied to detect neurogenesis in the DG region at day 14 and 28. In in vitro experiments, cell proliferation was detected by CCK8 and immunofluorescence, and the expression of cAMP/CREB signaling pathway-related proteins was detected by ELISA assay and Western blot.

**Results:**

CGP significantly improved spatial learning and memory disorders caused by CI, and it enhanced the proliferation of neural stem cells (NSCs), the number of immature neurons, and the differentiation from newborn cells to neurons. In vitro experiments further confirmed that CGP dose-dependently enhanced the cell viability of NSCs, and immunofluorescence staining showed that CGP promoted the proliferation of NSCs. In addition, treatment with CGP increased the expression of cAMP, PKA, and pCREB in cultured NSCs.

**Conclusion:**

Inhibition of GABA_B_ receptor can effectively promote hippocampal neurogenesis and improve spatial learning and memory in adult mice following CI.

## Introduction

Cerebral ischemia (CI) injury caused spatial learning and memory deficits, possibly due to the death of a large number of hippocampal pyramidal neurons [1–3]. On the other hand, accumulating evidence suggested that increasing neurogenesis after CI may contribute to the recovery of cognitive function [4–6]. The number of endogenous neurogenesis induced by CI is insufficient to fully restore brain function [4, 7, 8]. Therefore, it is meaningful to improve cognitive impairment caused by CI through promoting neurogenesis to supplement lost neurons.

Gamma aminobutyric acid (GABA), the main inhibitory neurotransmitter, plays an indispensable role in injury and repair of CI [9–12]. GABA works through two main types of receptors: ionotropic GABA_A_ receptor and metabotropic GABA_B_ receptor. Enhancement of signals acting on GABA_A_ receptor showed neuroprotective effects in CI animals [13, 14]. GABA_A_α5 receptor antagonists administered by oral gavage 72 h after stroke can enhance neural precursor cell proliferation and neuronal differentiation in the ipsilateral subventricular region of mice [15]. Moreover, intraperitoneal injection of GABA_A_ receptor inverse agonists on day 7 after stroke increased neuronal proliferation in the peri-infarct zone in rats [16].

GABA_B_ receptor affects progenitor proliferation and migration both in the developing brain [17] and in adult neurogenesis [18, 19]. Intracranial injection of GABA_B_ receptor antagonists in healthy adult mice promoted neural stem cells (NSCs) in the subgranular zone (SGZ) recruiting resting cells to active proliferative stem cell pool, and gene deletion of GABA_B1_ receptor subunit increased NSC proliferation in vivo [19]. Intraperitoneal injection of GABA_B_ receptor antagonists promoted hippocampal neurogenesis in depressed mice [18]. So far, the effect of inhibition of GABA_B_ receptor on neurogenesis after CI has not been reported. Increased GABA concentration in the dentate gyrus (DG) region was detected 30 days after permanent bilateral common carotid artery occlusion (BCCAO) in rats and further proved that activation of GABA_B_ receptors was related to cognitive impairment [10]. The DG region is also the main site of neurogenesis [20], which is involved in the process of learning and memory [21]. Since promoting hippocampal neurogenesis can improve cognitive impairments in CI mice [4], we speculated that GABA_B_ receptor antagonists may promote cognitive recovery after CI by enhancing neurogenesis.

The complex brain microenvironment plays an important role in the activation of mammalian NSCs [22]. Hippocampal neurogenesis can be regulated by directly stimulating NSCs or by indirectly altering microenvironment [23]. Conditional knockout of GABA_B1_ subunits of adult neural progenitor cells in the DG region of healthy mice enhanced neurogenesis [19]. We investigated the effects of CGP52432 (GABA_B_ receptor antagonists, CGP) on neurogenesis and cognition in CI models induced by transient BCCAO for 20 min and also conducted in vitro experiments to explore the role of CGP on the proliferation of NSCs and related mechanisms.

## Materials and methods

### Animals and experimental design

Adult male C57BL/6J mice (6–8 weeks old) purchased from the Experimental Animal Center of Chong Qing Medical University (Chongqing, China) were used in in vivo studies. All animal procedures complied with the Guide for the Care and Use of Laboratory Animals published by the US National Institutes of Health (NIH) and are also approved by the Institutional Animal Care and Use Committee at Chong Qing Medical University (License Number: SYXK 2018-0003). Mice were housed in a standardized environment with a humidity of 60–65% and controlled temperature at 23 ± 2 °C with a 12-h light–dark cycle.

**Fig. 1:**
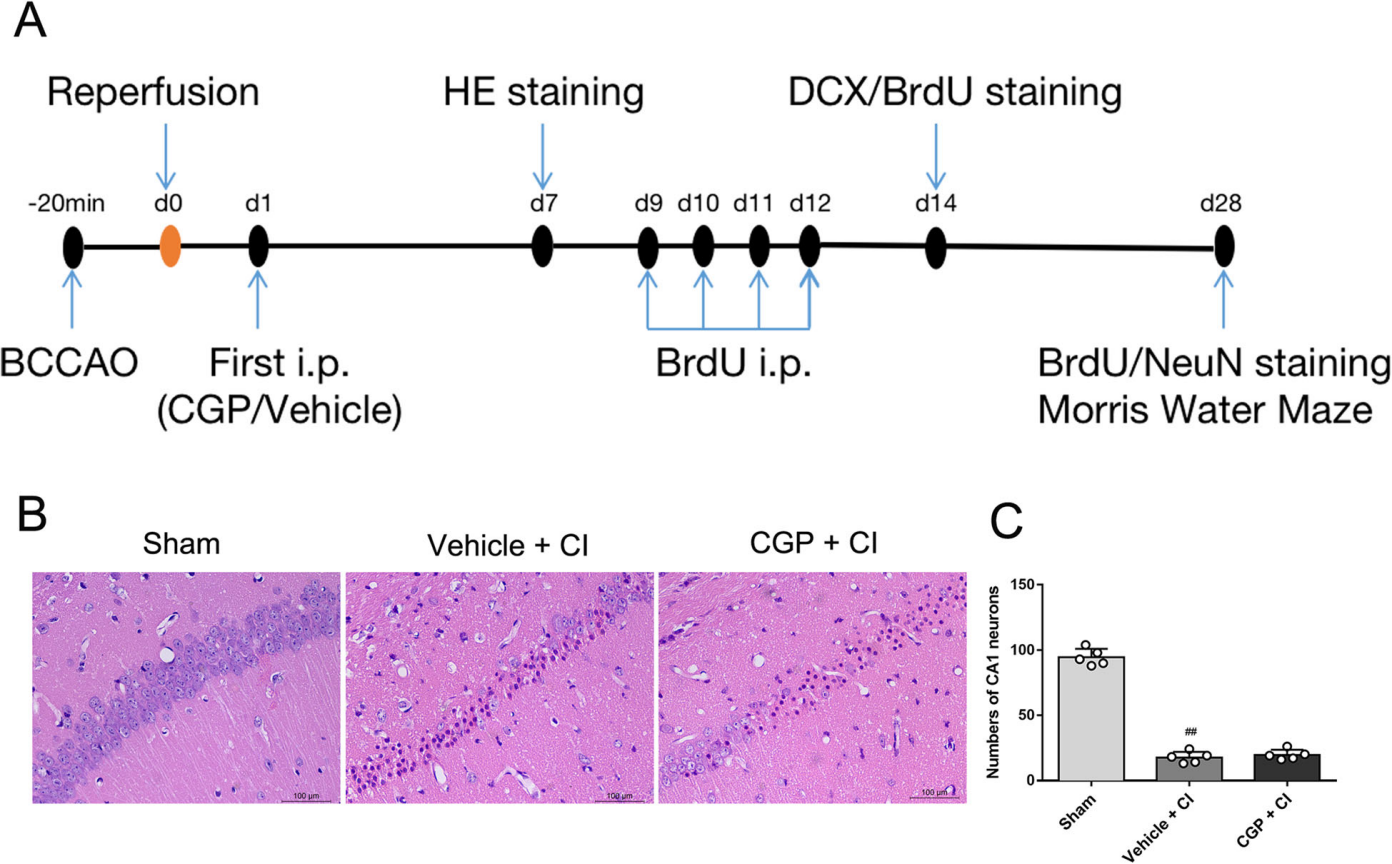
**a** The experimental protocol. The mouse CI model was induced by BCCAO for 20 min. Experimental mice were started to receive either CGP (10 mg/kg; i.p.) or an equal volume of vehicle (0.1 M PBS, i.p.) at day 1 after CI. HE staining was performed for morphological changes at days 7 after CI. BrdU (50 mg/kg; i.p.) was administrated daily for 4 consecutive days from 9 to 12 days after CI. And then mice were sacrificed for immunofluorescence staining (BrdU, DCX, NeuN) at 14 and 28 days after CI. Morris water maze (MWM) test was conducted to evaluate the spatial learning and memory abilities at days 28 after CI. **b**, **c** Effect of CGP on CI-induced histological changes in hippocampal CA1 neurons (*n* = 5 mice in each group). **b** Microphotographs of HE staining in hippocampal CA1 7 days after reperfusion (scale bar 100 μm). **c** Histogram showing the number of CA1 neurons in hippocampal (the data are expressed as the mean ± SD). ^##^*P* < 0.01 compared with the sham group (one-way ANOVA with Tukey post-test)

Experimental procedures for in vivo studies are described in Fig. 1a. Mice were randomly assigned to three groups: the sham group, the vehicle + CI group, and the CGP + CI group (CGP52432 is an antagonist of GABA_B_ receptor, CGP), 21 mice in each group. Animal exclusion criteria include sobbing-like breath, arterial rupture or serious infection, and death during operation or after surgery. To minimize mouse suffering, qualified and experienced laboratory staff will handle these mice with care. Mice were placed on a thermostatic blanket after surgery to keep them warm. The general condition of mice is closely monitored daily.

### Drug treatment

CGP52432 (MCE, Cat# HY-103531) was dissolved in phosphate-buffered saline (PBS, as a vehicle) and diluted to 1.0 mg/ml. Mice in the CGP + CI group were subjected to 20 min BCCAO and received daily intraperitoneal (i.p.) injection of CGP52432 (10 mg/kg) starting 24 h after CI for 7 days or 14 days. The sham group and the vehicle + CI group were intraperitoneally injected with equal volumes of PBS.

For neurogenesis analysis, all mice were treated with daily 50 mg/kg i.p. injections of 5-bromo-2′-deoxyuridine (BrdU, Sigma-Aldrich, Cat# B5002) for 4 consecutive days from days 9 to 12 after surgery. All mice were sacrificed on days 14 and 28, and tissues were collected for further immunofluorescence staining.

### Transient cerebral ischemic

Mice were anesthetized with 3.5% chloral hydrate (350 mg/kg, i.p.), and CI was induced by transient BCCAO for 20 min, as described previously [4, 24]. Sham-operated mice were treated with the same surgical procedure, except for carotid occlusion. The body temperature of the animals was maintained at 37 ± 0.5 °C using a thermostat until the mice resuscitated.

### Morris water maze test

Spatial learning and memory performance were measured at day 28 using the Morris water maze (MWM) according to previously published methods. The maze consisted of a large circular pool (120 cm in diameter, 45 cm in height, filled to a depth of 30 cm with water at 28 ± 1 °C) and divided into four equal-sized quadrants. Water was made opaque with milk. A white platform (5 cm in diameter) was submerged and placed 1 cm below the surface of the water in the center of the target quadrant to provide an escape zone.

In order to assess the ability of spatial learning, all mice were allowed 4 training trials each day for 5 consecutive days. The position of the platform was maintained unaltered throughout the training session. During the place navigation trial, the mice swam freely into the water along the four-quadrant pool wall in turn. Each mouse was allowed a maximum of 60 s to find the submerged white platform. The time of the mice to find the hidden platform was recorded as the escape latency time. If the mouse fails to find the platform within 60 s, it is manually guided to the platform and allowed to rest for 30 s and then the escape latency time is recorded as 60 s. The escape latency time to find the hidden platform and swim velocity were recorded. To assess spatial memory ability, mice were subjected to a 60-s spatial probe trial on day 6 in which the platform was removed. The numbers of crossing original platform’s location were thought to reflect spatial memory capabilities. The number of entries to platform location and swim velocity were recorded. All behaviors of the mice were collected by a digital camera located directly above the water maze and the data fed back to the computer was analyzed.

### Tissue preparations and hematoxylin and eosin staining

To detect changes in the morphology and number of pyramidal neurons in the cornu ammonis area 1 (CA1), hematoxylin and eosin (HE) staining was performed. Five mice in each group were sacrificed for HE staining on the 7th day. Animals were deeply anesthetized with chloral hydrate and transcardially perfused with 0.1 M PBS and then with 4% paraformaldehyde. The brains were fixed in 4% paraformaldehyde at 4 °C and followed by paraffin embedding. The coronal serial sections (− 1.3 to − 2.3 mm from Bregma) were cut into a thickness of 4 μm and prepared for HE staining. Every 10th section was stained and analyzed. Brain sections were deparaffinized, dyed with hematoxylin for 2 min, differentiated with 1% hydrochloric alcohol for 5 s, and then stained with eosin for 2 min. The sections were mounted with neutral resin and then covered with a coverslip. In each section, three micrograph areas (at high power × 40 objective) of the CA1 region were randomly selected for quantification of neurons. Cells with obvious nucleus and nucleolus were counted. The mean number of pyramidal cells in the micrograph area of the CA1 region of each mouse was calculated for statistical analysis.

### Tissue preparations and immunofluorescence

The peak of neurogenesis after CI is 1–2 weeks after ischemia [25, 26], and the newly generated neural cells mature into functional neurons integrated into the neural network at 4 weeks [27]. Therefore, BrdU and BrdU/NeuN immunofluorescence staining were performed on the 14th and 28th days after CI, respectively. At 14 or 28 days after CI reperfusion, mice were deeply anesthetized with chloral hydrate and transcardially perfused with PBS and then with 4% paraformaldehyde. Brain tissue was then removed and fixed in 4% paraformaldehyde at 4 °C and dehydrated with a gradient concentration sucrose solution. And then coronal serial sections (− 1.3 to − 2.3 mm from Bregma) in 12-μm thicknesses were cut and stored at − 80 °C until use. Every 8th section was stained and analyzed. For BrdU and BrdU/NeuN immunofluorescence, brain tissue sections were incubated in 2 N HCl for 30 min at 37 °C to break open the DNA structure of the labeled cells. Immediately, 0.1 M borate buffer was added to neutralize HCl. Next, all sections were permeabilized with 0.3% Triton X-100 for 30 min at 37 °C and then incubated with 5% goat serum (Boster, China, Cat#AR0009) for 2 h at room temperature. All sections were then incubated with 1:200 rat anti-BrdU (Abcam, UK, Cat# ab6326) and 1:200 rabbit anti-NeuN (Abcam, UK, Cat#ab177487) antibody overnight at 4 °C. For DCX (Doublecortin) immunofluorescence staining, except for HCl and borate buffer, the rest of the procedure is the same as BrdU immunofluorescence staining. Then, the sections were incubated with 1:200 rabbit anti-DCX (Abcam, UK, Cat# ab207175) antibody at 4 °C overnight. After washing in PBS, sections were incubated in 1:200 fluorescence-labeled secondary antibodies [goat anti-rat conjugated to DyLight 594 (Abbkine, USA, CAT#A23440) or goat anti-rabbit conjugated to DyLight 488 (Abbkine, USA, CAT#A23220)] at room temperature for 2 h. And then, sections were incubated with DAPI (Beyotime, China, CAT#C1005) for 5 min at room temperature to stain cell nuclei. Finally, 50% glycerol (Sigma-Aldrich, Cat#G5516) was applied and fixed with a cover slip and analyzed by immunofluorescence confocal microscopy (ZEISS, Germany). For BrdU and DCX immunofluorescence, the mean number of BrdU + cells and DCX + cells in the DG region of each section of each mouse was calculated for statistical analysis. For BrdU/NeuN immunofluorescence, the ratio of BrdU + NeuN + cells to BrdU + cells and the percentage of BrdU + NeuN + cells in each group to that in the sham group in the DG region of each mouse section was calculated for statistical analysis. Numbers of positive cells were counted using Image-Pro Plus 6.0 software.

### In vitro experiment

#### Cell culture and drug administration

Following the previous study, NSCs were extracted from neonatal 1–3-day-old Sprague-Dawley rats [28, 29]. Cell suspension was plated at a density of 1 × 106 cells/ml in 25-cm^2^ cell culture flasks using DMEM/F12 medium supplemented with 2%B-27 (Thermo Fisher, USA, CAT#12587010), 20 ng/ml bFGF (Peprotech, CAT#400-29), 20 ng/ml EGF (Peprotech, CAT#400-25), and penicillin and streptomycin. The resultant neurospheres were harvested and dissociated to single cell suspension for replating every 7 days. All experiments were performed during the third passage. In the in vitro cell culture experiment, the experimental group (CGP group) was treated with CGP for 24 h, and the vehicle group was treated with the corresponding volume of PBS.

#### Immunofluorescence

In order to identify NSCs, neurospheres and single cells were plated on culture plates which placed polylysine-coated coverslips. Neurospheres or single cells were fixed with 4% paraformaldehyde. Neurospheres and single cells were permeabilized with 0.3% Triton X-100 for 30 min at 37 °C and then incubated with 5% goat serum (Boster, China, Cat#AR0009) for 2 h at room temperature. Then, the neurospheres and single cells were incubated with 1:100 rabbit anti-Nestin (Abcam, UK, Cat# ab6142) antibody overnight at 4 °C. After washing in PBS, they were incubated in 1:200 fluorescence-labeled secondary antibodies [goat anti-mouse conjugated to DyLight 488 (Abbkine, USA, CAT#A23210)] at room temperature for 2 h. And then, cells were incubated with DAPI (Beyotime, China, CAT#C1005) for 5 min at room temperature to stain cell nuclei. Finally, the neurospheres and single cells were analyzed by immunofluorescence confocal microscopy (ZEISS, Germany).

To evaluate the proliferation of NSCs, single cells were plated on culture plates which placed polylysine-coated coverslips. Single NSCs were incubated with 10 μM BrdU and 1 μM CGP or equal volume PBS for 24 h. NSCs were permeabilized with 0.3% Triton X-100 for 30 min and then incubated in 2N HCl for 30 min at 37 °C to break open the DNA structure of the labeled cells. Immediately, 0.1 M borate buffer was used to neutralize HCl. Then, NSCs were incubated with 5% goat serum (Boster, China, Cat#AR0009) for 2 h. After that, NSCs were incubated with 1:200 BrdU antibody (Abcam, UK, Cat#ab6326) overnight at 4 °C. Cells were incubated with 1:200 fluorescence-labeled secondary antibodies [goat anti-mouse conjugated to Dylight 594 antibody (Abbkine, USA, CAT#A23410)] for 2 h at room temperature and then incubated with DAPI for 5 min. Finally, the cells were analyzed by immunofluorescence confocal microscopy (ZEISS, Germany) and numbers of positive cells were counted with Image-Pro Plus

 6.0 software.

#### CCK-8 assay and ELISA assay

The Cell Counting Kit-8 (CCK-8 assay) is used for the analysis of cell proliferation. In short, NSCs (approximately 5 × 104 cells/ml) of the 3rd passage were seeded on the PLL-coated 96-well plate. According to the groups, treatment with different concentrations of CGP for 24 h, 1/10 volume of CCK8 (Genview, CAT#GK3607) solution was added to each well, and after 4 h of incubation, the optical density (OD) values at 450 nm were measured with a microplate reader (Thermo).

Cells were rinsed twice with PBS, lysed in RIPA lysis buffer (Beyotime, China, CAT#P0013B), and protein concentration was measured using the BCA reagent (Beyotime, China, CAT#P0012). cAMP levels per unit protein were detected using the ELISA kit according to the manufacturer’s instructions. A standard curve was established based on the OD values measured at 450 nm on a microplate reader to calculate the concentration of cAMP.

#### Western blot analysis

Cells were rinsed twice with PBS, lysed in RIPA lysis buffer (Beyotime, China, CAT#P0013B) supplemented with a protease inhibitor (PMSF, Beyotime, China, CAT#ST506) and Phosphatase inhibitor cocktail A (Beyotime, China, CAT#P1082), and protein concentration was measured using the BCA reagent (Beyotime, China, CAT#P0012). Electrophoresis was conducted by 10% SDS-polyacrylamide gel and transferred proteins to PVDF membranes (Millipore, Cat# IPVH00010). The following primary antibodies were used for incubation overnight at 4 °C: 1:10,000 mouse anti-beta-Tubulin (Proteintech, China Cat#66240-1-Ig), 1:1000 rabbit anti-PKA (Cell Signaling, USA Cat#5842 T), 1:5000 rabbit anti-pCREB (Abcam, UK Cat# ab32096), and 1:1000 rabbit anti-CREB (Abcam, UK Cat# ab32515). All membranes were incubated with HRP-conjugated Affinipure Goat Anti-Mouse IgG (H+L) (Proteintech, China Cat#SA00001-1) or HRP-conjugated Affinipure Goat Anti-Rabbit IgG (H+L) (Proteintech, China Cat#SA00001-2) for 1 h. Immunoreactive bands were detected using an enhanced chemiluminescence (ECL) kit (Advansta, USA Cat#K-12045-D10) and quantified with a gel-image analyzing system (Fusion Optix, USA).

### Statistical analysis

All data were analyzed using GraphPad Prism 7.0 (GraphPad, USA) software. Statistical comparisons were conducted by one-way or two-way repeated measures ANOVA with Tukey post hoc testing or unpaired Student’s *t* test with Kolmogorov–Smirnov test. A *P* value of less than 0.05 was considered statistically significant (*P* < 0.05), and a *P* value of less than 0.01 was considered statistically highly significant (*P* < 0.01).

## Result

### Histopathological changes in the hippocampal CA1 region after CI

As shown in Fig. 1b, HE staining revealed that the pyramidal neurons had a clear structure, closely arranged, and the nucleolus was round, and the staining was clear with dark blue in the hippocampal CA1 region of the sham group. On the contrary, in the vehicle + CI group and the CGP + CI group, the neurons in the hippocampus CA1 region revealed significant damaged, with obvious nuclear contraction, irregular morphology, and loose arrangement. Compared with the sham group, the number of neurons was significantly reduced in the CA1 region of the vehicle + CI group, indicating that the model was reliable (Fig. 1c; *P* < 0.01). There was no significant difference in the number of neurons in the CA1 region between the CGP + CI group and the vehicle + CI group (Fig. 1c; *P* > 0.05).

### CGP improves spatial learning and memory deficits induced by CI

**Fig. 2:**
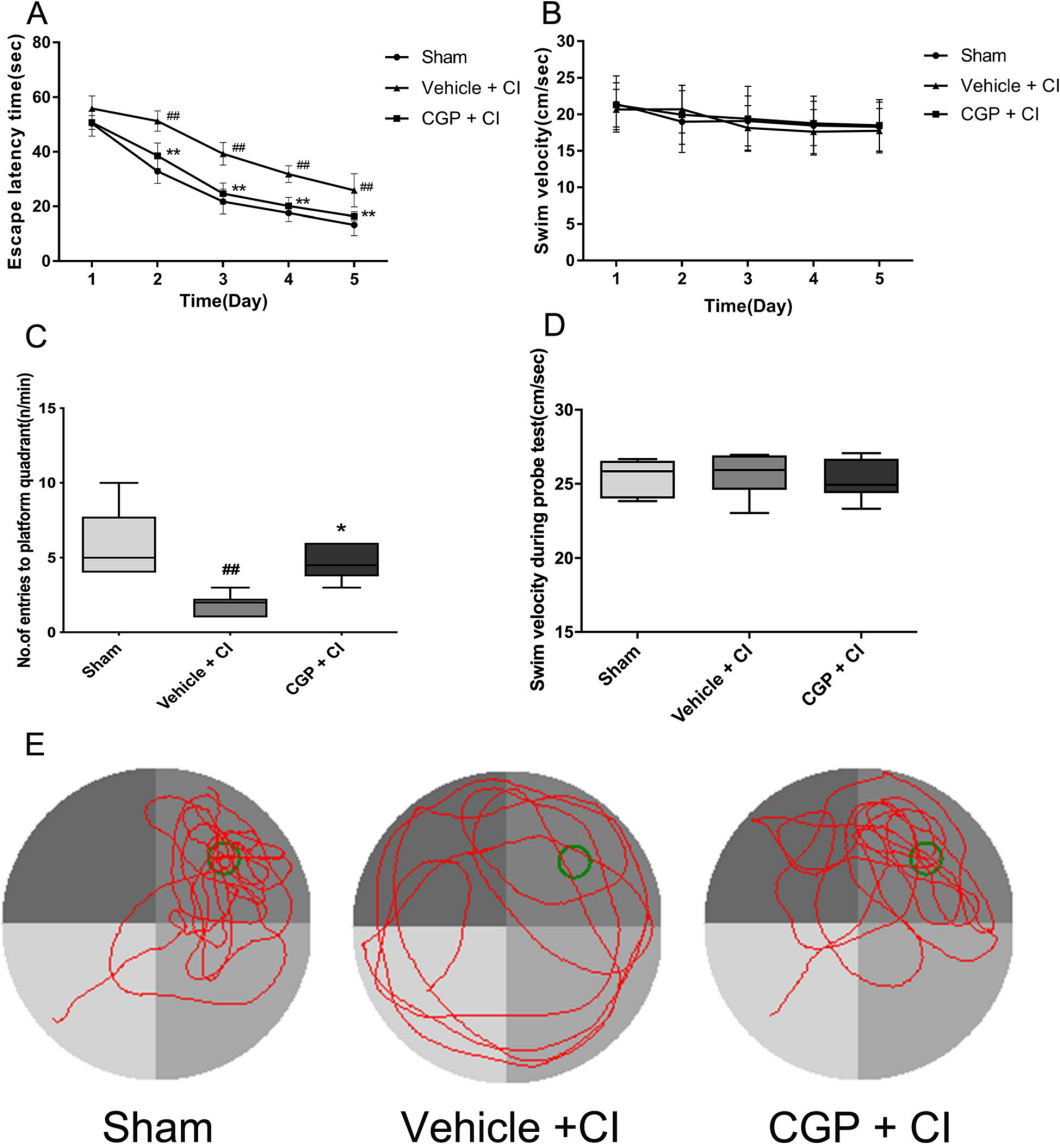
CGP facilitates cognitive recovery following CI (*n* = 6 mice in each group). **a** Escape latency to find the hidden platform in the place navigation test (the data are expressed as the mean ± SD). **b** No difference in swimming velocity was observed between the three groups in the place navigation test (the data are expressed as the mean ± SD). **c** Number of entries to platform quadrant in the spatial probe trial. **d** No difference in swimming velocity was observed between the three groups in the spatial probe trial. In this box and whisker plot (**c**, **d**), the line is the median, the ends of the box represent the upper and lower quartiles, and the whiskers represent the highest and lowest points. ^##^*P* < 0.01 compared with the sham group; *P < 0.05, **P < 0.01 compared with the vehicle + CI group. **e** The typical trajectories of mice in different groups in the spatial probe trial. Green circle indicates the location of the platform. Each mouse was performed to a 60-s probe test, where the platform was removed from its original location. The swimming trajectory shows that the mice of the vehicle + CI group explored all quadrants, while the mice of the sham group and CGP + CI group spend most of the time exploring target quadrants (two-way ANOVA with Tukey post-test)

In the Morris water maze test, the escape latency of the place navigation trial, the number of times the mice crossed the platform during the spatial probe test, and the swimming speed of the mice are showed in Fig. 2. In the place navigation trial, the escape latency time of each group of mice was longer at the beginning of the training period, and the escape latency time was correspondingly shortened as the training times increased. On the first day of training, compared with the sham group, the mice in the vehicle + CI group took longer to find the platform, but there was no statistical significance (Fig. 2a; *P* > 0.05). On days 2–5 of training, compared with the sham group, the time to escape latency of the vehicle + CI group was significantly increased (Fig. 2a; *P* < 0.01), indicating that CI caused impairment of learning; compared with the vehicle + CI group, the time to escape latency of the CGP + CI group was significantly shortened (Fig. 2a; *P* < 0.01), indicating that CGP administration improved learning impairment caused by CI. In the spatial probe trial, compared with the sham group, the number of crossings over the platform location in the vehicle + CI group significantly reduced (Fig. 2c; *P* < 0.01), indicating that CI caused a significant decrease in memory ability of the mice; compared with the vehicle + CI group mice, the number of times that the CGP + CI group crossed the platform location within a specified time significantly increased (Fig. 2c; *P* < 0.05), indicating that memory ability was significantly improved in CGP + CI group mice. In the place navigation and the spatial probe trial, the swimming velocity of the mice in each group has no significance (Fig. 2b, d; *P* > 0.05). Figure 2e shows typical trajectories of spatial probe test in each group.

### CGP promoted ischemia-induced neurogenesis in the hippocampal DG region

**Fig. 3:**
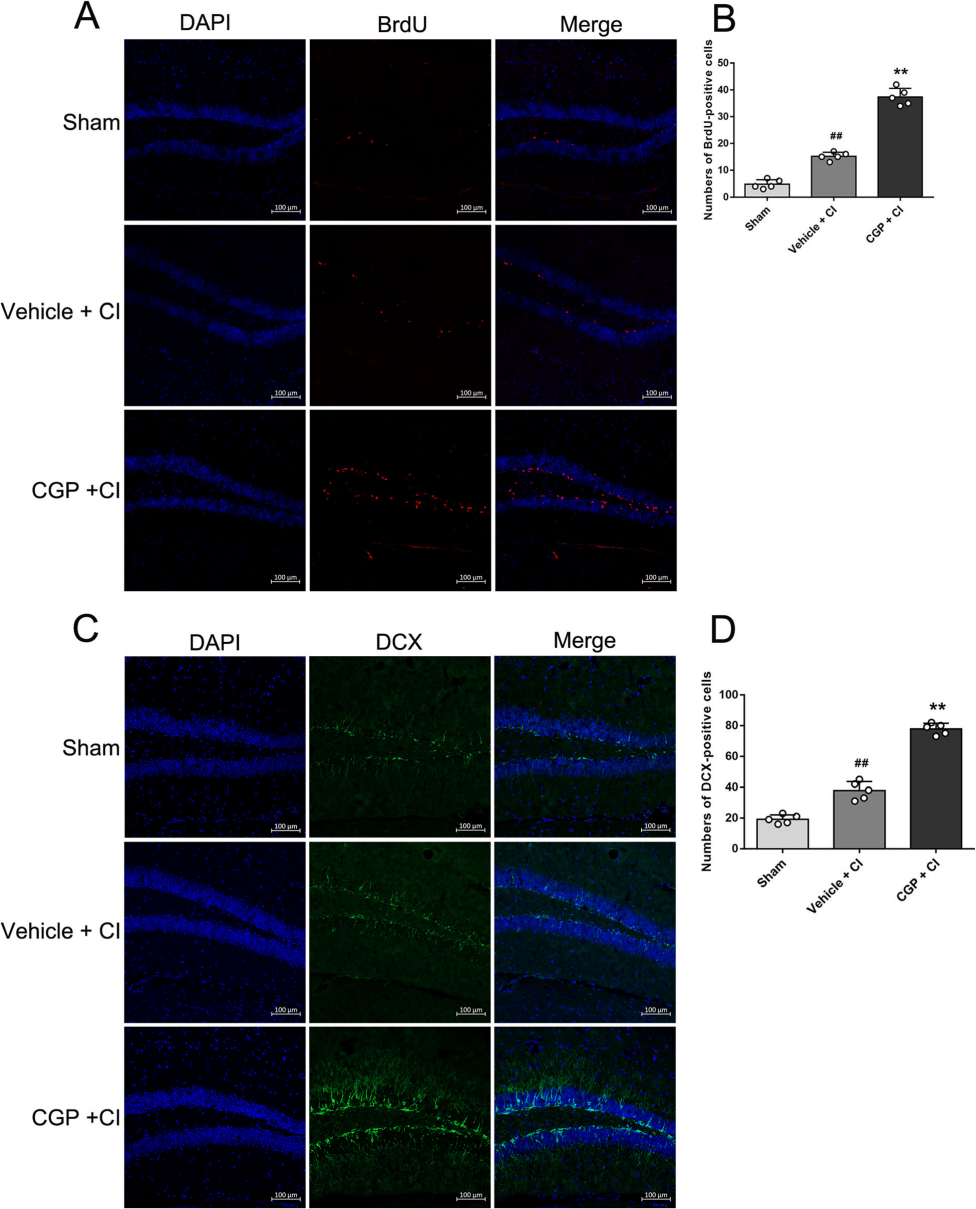
CGP promotes neurogenesis in the hippocampal DG (*n* = 5 mice in each group). **a** The confocal microscopic images showing BrdU (red)-positive cells in the hippocampal DG of each group 14 days after reperfusion (scale bar 100 μm). **b** Histograms showing the number of BrdU-positive cells in the hippocampal DG (the data are expressed as the mean ± SD). ^##^*P* < 0.01 compared with the sham group; **P < 0.01 compared with the vehicle + CI group. **c** The confocal microscopic images showing DCX (green)-positive cells in the hippocampal DG of each group 14 days after reperfusion (scale bar 100 μm). **d** Histograms showing the number of DCX-positive cells in the hippocampal DG (the data are expressed as the mean ± SD). ^##^*P* < 0.01 compared with the sham group; **P < 0.01 compared with the vehicle + CI group (one-way ANOVA with Tukey post-test)

To investigate the effect of CGP on the proliferation of the NSCs following CI, BrdU^+^ cells in the hippocampal DG region were counted (Fig. 3a). On the 14th day after CI, the number of BrdU^+^ cells in the vehicle + CI group increased compared with the sham group (Fig. 3b; *P* < 0.01). Compared with the vehicle + CI group, the number of BrdU^+^ cells significantly increased in the CGP + CI group (Fig. 3b; *P* < 0.01). DCX is a marker of adult neurogenesis and can be used to identify immature neurons [30, 31]. Figure 3c shows the immunofluorescence staining of DCX at 14 days after CI. Compared with the sham group, the number of DCX^+^ cells in the hippocampal DG region increased in the vehicle + CI group (Fig. 3d; *P* < 0.01). Compared with the vehicle + CI group, the number of DCX^+^ cells in the DG region significantly increased in the CGP + CI group (Fig. 3d; *P* < 0.01).

**Fig. 4:**
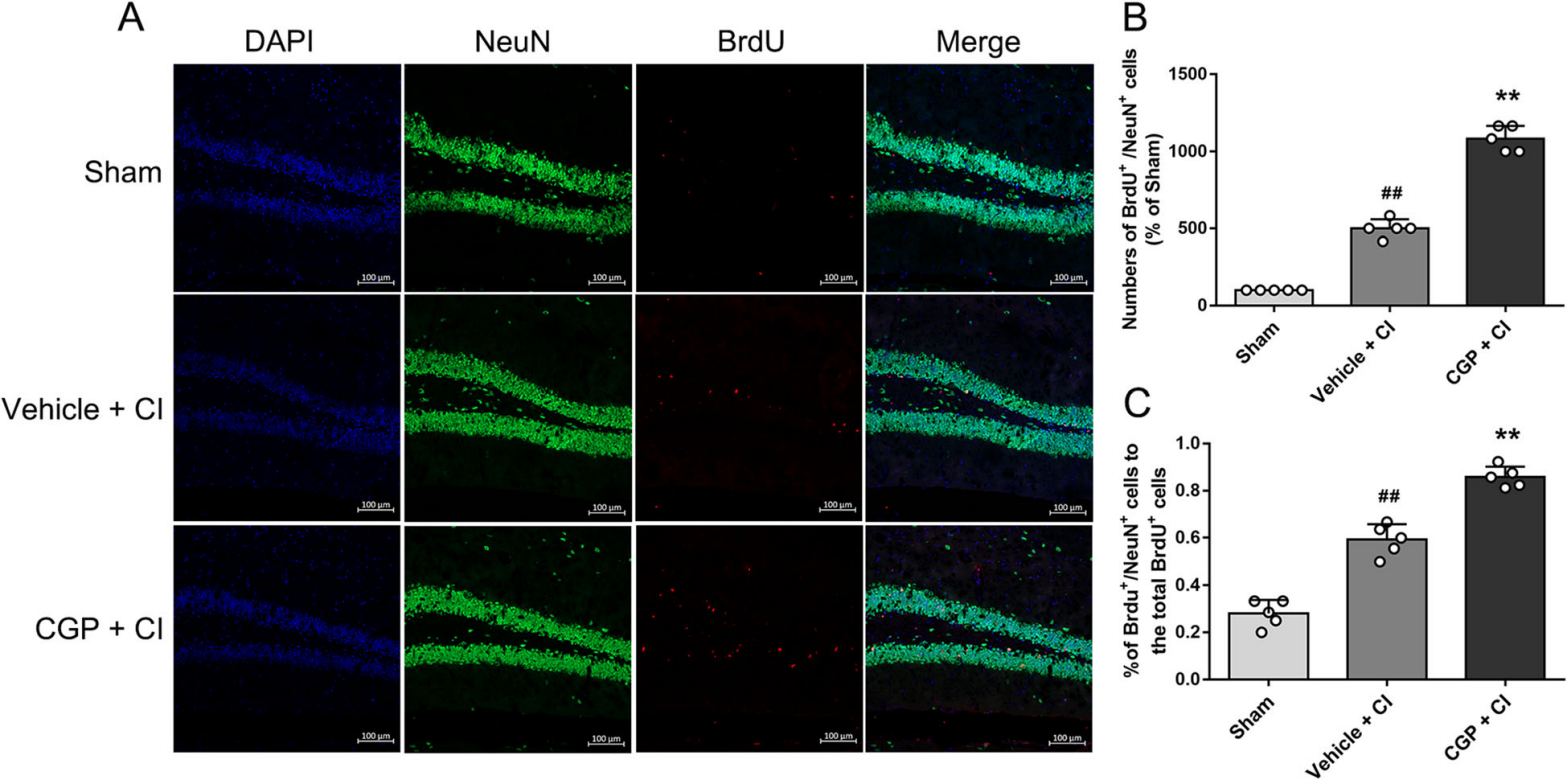
CGP promotes the differentiation of newborn cells into neurons (*n* = 5 mice in each group). **a** The confocal microscopic images showing BrdU (red) and NeuN (green) double-stained cells in the hippocampal DG of each group 28 days after reperfusion (scale bar 100 μm). **b** Histograms showing the number of BrdU+/NeuN+ cells in the hippocampal DG (the data are expressed as the mean ± SD). **c** Histograms showing the percentage of BrdU+/NeuN+ cells to the total BrdU+ cells (the data are expressed as the mean ± SD). ^##^*P* < 0.01 compared with the sham group; **P< 0.01 compared with the vehicle + CI group (one-way ANOVA with Tukey post-test)

To study the effect of CGP on the differentiation of NSCs following CI, BrdU and NeuN were double labeled on the 28th day after CI (Fig. 4a). Compared with the sham group, the number of BrdU^+^/NeuN^+^ cells increased in the vehicle + CI group (Fig. 4b; *P* < 0.01). In addition, compared with the vehicle + CI group, the number of BrdU^+^/ NeuN^+^ cells in the CGP + CI group significantly increased (Fig. 4b; *P* < 0.01), indicating the survival of the majority of CGP-induced new neural cells. The percentage of BrdU^+^/NeuN^+^ cells to the total BrdU^+^ cells increased compared with the sham group (Fig. 4c; *P* < 0.01). Compared with the vehicle + CI group, the percentage of BrdU^+^/NeuN^+^ cells to the total BrdU^+^ cells significantly increased in the CGP + CI group (Fig. 4c; *P* < 0.01), indicating that the differentiation of new neural cells into neurons is increased.

### CGP enhanced the proliferation of NSCs cultured in vitro

**Fig. 5:**
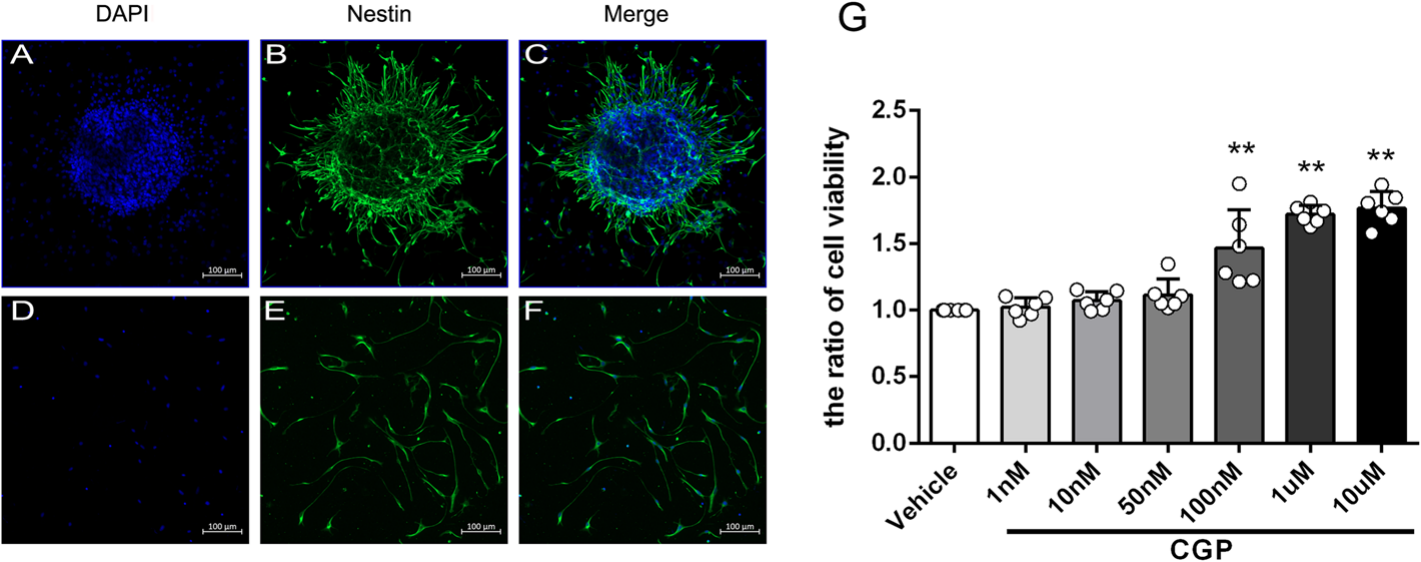
Identify NSCs and the effect of CGP on cell viability of NSCs. **a**–**c** The confocal microscopic images of neurosphere that stained with nestin (green) and counterstained with DAPI (blue) as the nuclear marker (scale bar 100 μm). **d**–**f** The confocal microscopic images of single neural cells stained with nestin (green) and counterstained with DAPI (blue) as the nuclear marker (scale bar 100 μm). **g** The histogram showing the cell viability values detected by the CCK8 assay for each group. **P < 0.01 compared with the vehicle group; ^#^*P* < 0.05, ^##^*P* < 0.01 compared with the 100 nM CGP group (the data are expressed as the mean ± SD, *n* = 6 samples per condition; unpaired Student’s *t* test with Kolmogorov–Smirnov test)

**Fig. 6:**
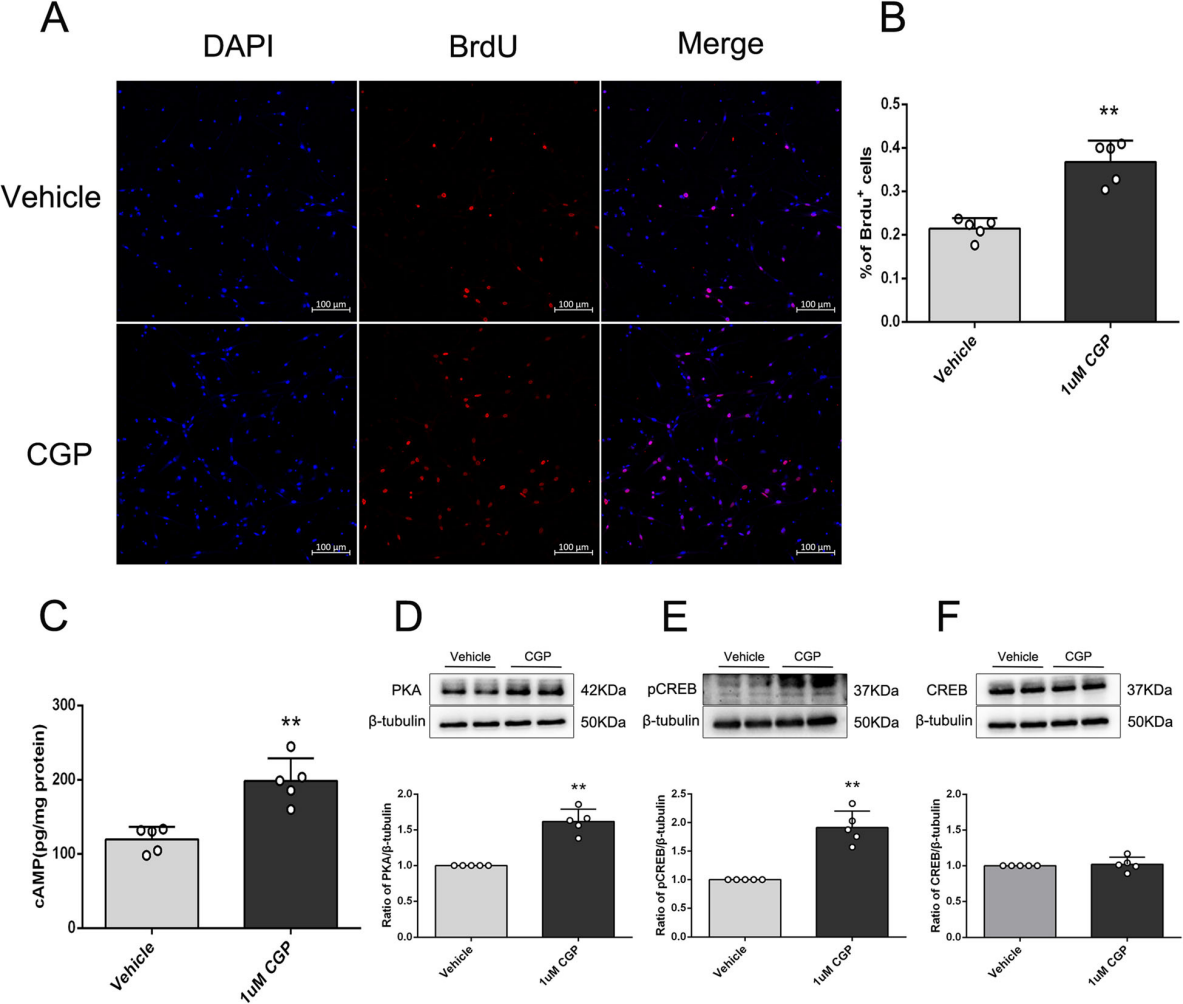
CGP promotes the proliferation of NSCs and activates the cAMP–CREB signaling pathway in vitro. **a** The confocal microscopic images of BrdU (red)/DAPI (blue) stained NSCs after incubation with vehicle or CGP for 24 h (scale bar 100 μm). **b** Histograms showing the quantitation of BrdU-positive cells (the data are expressed as the mean ± SD, *n* = 5 samples per condition). **P < 0.01 compared with the vehicle group. **c** The histogram shows the cAMP levels detected by the ELISA assay. **d**–**f** (upper) Representative bands of cAMP, PKA, pCREB, and CREB. **d**–**f** (lower) Expression of PKA, pCREB, and CREB in each group determined by Western blot. Western blot quantitation was performed using densitometric analysis (the data are expressed as the mean ± SD, *n* = 5 samples per condition). **P < 0.01 compared with the vehicle group (unpaired Student’s *t* test with Kolmogorov–Smirnov test)

Due to the complex microenvironment of the brain system, in order to study the direct effect of CGP on neural stem cells and its underlying mechanism, in vitro experiments were also conducted. As shown in Fig. 5a–f, both neurospheres and single cells express Nestin (Nestin is a marker of neural stem cells). Nestin staining of single cells showed that the purity of neural stem cells was > 95%. After treatment with different concentrations of CGP for 24 h, the cell viability of the 100-nM, 1-μM, and 10-μM groups was significantly increased compared with the vehicle group (Fig. 5g; *P* < 0.01). Compared with the 100-nM group, the cell viability of the 1-μM and 10-μM groups was increased, and there was no statistical difference between the 1-μM group and 10-μM group (Fig. 5g; *P* < 0.01). As shown in Fig. 6a, b, the percentage of BrdU+ cells in the 1-μM CGP group was higher compared with the vehicle group (*P* < 0.01). This result showed that CGP induced neural stem cell proliferation.

### CGP promoted neurogenesis by cAMP/CREB pathway

The cAMP/CREB signaling pathway has been implicated in the mechanism of neurogenesis [32]. To investigate the mechanism, the activation of the cAMP/CREB pathway was explored using 1-μM CGP treatment. As shown in Fig. 6c, the results of ELISA showed that the cAMP concentration in the 1-μM CGP group was significantly higher than that in the vehicle group. The results of Western blot showed that the protein expression levels of PKA and pCREB were significantly increased compared with the vehicle group (Fig. 6d–f; *P* < 0.01). These results indicated that 1 μM CGP activated the cAMP/CREB signaling pathway.

## Discussion

In the present study, we found that CGP treatment had significant effects on improving spatial learning and memory disorders in adult mice subjected to BCCAO. Furthermore, CGP was demonstrated to promote NSCs of the hippocampal DG proliferation and differentiation to neuron in CI mice. The vitro study further showed that the inhibition of GABA_B_ receptor by CGP has the ability to enhance NSC proliferation.

In this study, learning and memory dysfunction was observed in adult mice following CI by MWM testing, which is consistent with previous reports [1, 3, 4, 33, 34]. There was no significant difference in the swimming speed of each group of animals in the MWM testing, and they showed the equivalent exercise ability. In this study, the treatment of CGP after 24 h of BCCAO significantly improved the performance of mice in place navigation trial and space exploration experiment. The hippocampus is an important physiological structure involved in spatial learning and memory [35]. In our experiment, as mentioned in previous studies, transient BCCAO mainly damaged pyramidal neurons in the hippocampal CA1 region [1, 4, 24, 36–38]. Specifically, the morphological structure of neurons changed and the number of surviving neurons decreased after acute CI. Relative to the acute ischemia neuroprotective effect of GABA_A_ receptor activation, there were several partly confusing reports of the protective effects of GABA_B_ receptor [39–43]. Immediate activation of GABA_B_ receptor during glucose-oxygen deprivation showed neuroprotection against ischemia in organo-typic hippocampal slices [39] and intraperitoneal injection of GABA_B_ receptor agonists 2 weeks after permanent BCCAO reduced hippocampal neuron damage in rats [40]. An increase in the expression of GABA_B_ receptor by acupuncture or Chinese medicine given as soon as the rats recovered from reperfusion can reduce the cerebral infarct volumes in focal cerebral ischemia/reperfusion injury rats [41, 42]. However, inhibition of GABA_B_ receptors 1 day after surgery reduced neuron death in the hippocampal CA1 region caused by surgical trauma [43]. GABA_B_ receptor antagonist injection in the hippocampal DG region on day 30 after permanent BCCAO in rats improved cognition [10], suggesting that GABA_B_ receptor antagonist was administrated at the late stage of ischemia may exert neuroprotective effects, or at least not exacerbate ischemic neuron damage. The above studies showed that activating GABA_B_ receptor immediately after ischemic injury has a neuroprotective effect, indicating that inhibition of GABA_B_ receptors in the early stages of CI may show the opposite effect. Therefore, GABA_B_ receptor antagonist was administrated 24 h after CI to avoid possible adverse reactions. In our study, there was no significant difference in the number of alive neurons in the CA1 region between the CGP + CI group and the vehicle + CI group, which indicates that the administration of GABA_B_ receptor antagonist 24 h after CI is safe, at least without aggravating ischemia neuron damage.

Neurogenesis is an extremely complex process, including neural stem cell proliferation, differentiation, survival, and integration. Adult neurogenesis mainly occurs in the subgranular zone of the hippocampus DG region and subventricular zone of the lateral ventricle in the adult mammalian brain [44, 45]. Additionally, adult hippocampus neurogenesis is related to learning and memory [21, 46–49]. CI promoted a certain degree of endogenous neurogenesis [25, 50], but only a small number of newly generated neural cells can survive and mature into functional neurons [4, 33]. In this study, CGP treatment significantly increased BrdU^+^ cells and DCX^+^ cells in the hippocampal DG region of CI mice on the 14th day, indicating that CGP has a significant pro-proliferative effect. The newly generated granular neurons in the DG region of the hippocampus are functionally integrated into the hippocampal circuit at 4 weeks [27]. Furthermore, the DG region is thought to play an essential role in learning, memory, and spatial navigation tasks [46], and previous studies demonstrated that neurogenesis in the DG region of the hippocampus can rescue the ability of learning and memory [33, 51, 52]. Our further study found that the number of BrdU^+^/NeuN^+^ cells increased significantly on day 28, indicating that the number of newborn cells that survived and differentiated into neurons increased. Therefore, the improvement of cognitive impairment in adult CI mice after CGP administration may be attributed to the role of CGP in promoting neurogenesis.

GABA_B_ receptor is widely distributed in mammalian brain tissues, among which the cortex, hippocampus, thalamus, basal ganglia, and cerebellum are most distributed. CGP can penetrate the blood–brain barrier [18] and is a highly selective and effective GABA_B_ receptor antagonist [53, 54], with the term half maximal inhibitory concentration (IC50) of about 85 nM [53]. Our study did not observe the side effects of intraperitoneal injection of CGP, which is consistent with previous studies [18, 55]. CGP promoted hippocampal neurogenesis in depressed mice [18]. Other GABA_B_ receptor antagonists similar in structure to CGP have properties to promote hippocampal neurogenesis in healthy adult mice [19]. Adult neurogenesis is closely related to cognitive function [21, 46, 49]. GABA_B_ receptor antagonist injected into the DG region on day 30 after permanent BCCAO in rats improved spatial learning and memory impairment [10], which may also be related to neurogenesis, but no related studies have been done so far. Besides, CGP treatment enhanced long-term potentiation of the DG region in hippocampal slices of Ts65Dn mice, a genetic model of Down syndrome, and rescued cognitive deficits of Ts65Dn mice, during hippocampally mediated memory tasks [55].

Hippocampal neurogenesis can be converted into neurons by directly stimulating neural stem cells, or indirectly regulated by altering the microenvironment [23]. GABA, as a niche signal, maintains the resting status of adult dentate NSCs by activating GABA_A_ receptors on NSCs [56]. In this study, we focused on whether CGP can directly affect the GABA_B_ receptor of neural stem cells. To address whether CGP has a direct effect on neural stem cell proliferation, we cultured neural stem cells in vitro and identified the cultured cells with nestin marker staining. The current CCK8 assay results confirmed that CGP dose-dependently enhanced the cell viability of neural stem cells. Meanwhile, we noticed that when CGP was administered at a concentration of 1 μM, it had a stable enhancement of the cell viability of cultured neural stem cells. In addition, immunofluorescence staining showed that 1-μM CGP treatment promoted the proliferation of NSCs in vitro. In this study, in vitro cultured cell experiments confirmed that CGP can directly affect NSCs themselves to promote proliferation.

cAMP–CREB signaling pathway is reported as one of the key signal transduction pathways that regulate the proliferation and differentiation of NSCs in vivo and in vitro [32, 57, 58]. In vitro experiments proved that activation of the cAMP–CREB cascade was necessary and sufficient for maturation of newborn neurons [32]. Activation of the cAMP cascade by taking rolipram increased cell proliferation in the dentate gyrus of the hippocampus, and this effect was accompanied by activation of CREB phosphorylation [57]. In addition to increasing the proliferation and survival of these cells, the cAMP–CREB cascade also promoted the maturation of neonatal neurons in the adult hippocampus [57, 58]. Our study revealed that administration of CGP increased the levels of cAMP, PKA, and pCREB in neural stem cells cultured in vitro. Overall, these data showed the importance of the cAMP–CREB cascade in neurogenesis, and inhibition of GABA_B_ receptors by CGP promoting the proliferation of neural stem cells may be through the cAMP–CREB signaling pathway. However, it has not been confirmed in this study whether inhibition of GABA_B_ receptor which induced the proliferation of neural stem cells was caused solely by the cAMP–CREB signaling pathway. Therefore, further research is needed.

## Conclusions

The current study demonstrated that the inhibition of GABA_B_ receptors by CGP effectively promoted hippocampal neurogenesis and improved cognitive impairments in adult mice following CI. In addition, CGP promoted the proliferation of NSCs cultured in vitro, and its potential mechanism may be mediated through the cAMP–CREB signaling pathway. These findings suggested that inhibition of GABA_B_ receptors may have potential value for clinical treatment of CI.

## Data Availability

The datasets used and/or analyzed during the current study are available from the corresponding author on reasonable request.

## Abbreviations

BCCAO: Bilateral common carotid artery occlusion
BrdU: 5-Bromo-2′-deoxyuridine
CA1: Cornu ammonis area 1
CI: Cerebral ischemia
DG: Dentate gyrus
GABA: Gamma aminobutyric acid
MWM: Morris water maze
NeuN: Neuron-specific nuclear-binding protein
NSCs: Neural stem cells
SGZ: Subgranular zone

## References

[1] Zhang H-P, Yuan L-b, Zhao R-n (2010). Isoflurane preconditioning induces neuroprotection by attenuating ubiquitin-conjugated protein aggregation in a mouse model of transient global cerebral ischemia. Anesth Analg.

[2] Cheng O, Ostrowski RP, Wu B (2011). Cyclooxygenase-2 mediates hyperbaric oxygen preconditioning in the rat model of transient global cerebral ischemia. Stroke.

[3] Luo C, Ren H, Wan J-B (2014). Enriched endogenous omega-3 fatty acids in mice protect against global ischemia injury. J Lipid Res.

[4] Chen L, Song D, Chen B (2020). Activation of liver X receptor promotes hippocampal neurogenesis and improves long-term cognitive function recovery in acute cerebral ischemia-reperfusion mice. J Neurochem.

[5] Mangin G, Poittevin M, Charriaut-Marlangue C (2019). Glatiramer acetate reduces infarct volume in diabetic mice with cerebral ischemia and prevents long-term memory loss. Brain Behav Immun.

[6] Yuan L, Sun S, Pan X (2020). Pseudoginsenoside-F11 improves long-term neurological function and promotes neurogenesis after transient cerebral ischemia in mice. Neurochem Int.

[7] Dillen Y, Kemps H, Gervois P (2020). Adult neurogenesis in the subventricular zone and its regulation after ischemic stroke: implications for therapeutic approaches. Transl Stroke Res.

[8] Marques BL, Carvalho GA, Freitas EMM (2019). The role of neurogenesis in neurorepair after ischemic stroke. Semin Cell Dev Biol.

[9] Liu J, Wang L-N (2014). Gamma aminobutyric acid (GABA) receptor agonists for acute stroke. Cochrane Database Syst Rev.

[10] Li G, Lv J, Wang J (2016). GABAB receptors in the hippocampal dentate gyrus are involved in spatial learning and memory impairment in a rat model of vascular dementia. Brain Res Bull.

[11] Mayor D, Tymianski M (2018). Neurotransmitters in the mediation of cerebral ischemic injury. Neuropharmacology.

[12] Feng Y-W, Huang Y-Q, Yan Y (2020). Phasic GABA signaling mediates the protective effects of cTBS against cerebral ischemia in mice. Neurosci Lett.

[13] Ginsberg MD (2008). Neuroprotection for ischemic stroke: past, present and future. Neuropharmacology.

[14] Brickley SG, Mody I (2012). Extrasynaptic GABA(A) receptors: their function in the CNS and implications for disease. Neuron.

[15] Wang Y-C, Dzyubenko E, Sanchez-Mendoza EH (2018). Postacute delivery of GABA α5 antagonist promotes postischemic neurological recovery and peri-infarct brain remodeling. Stroke.

[16] He W-M, Ying-Fu L, Wang H (2019). Delayed treatment of α5 GABAA receptor inverse agonist improves functional recovery by enhancing neurogenesis after cerebral ischemia-reperfusion injury in rat MCAO model. Sci Rep.

[17] Fukui M, Nakamichi N, Yoneyama M (2008). Modulation of cellular proliferation and differentiation through GABA(B) receptors expressed by undifferentiated neural progenitor cells isolated from fetal mouse brain. J Cell Physiol.

[18] Felice D, Pizzo RC, O'Leary OF (2012). Blockade of the GABA(B) receptor increases neurogenesis in the ventral but not dorsal adult hippocampus: relevance to antidepressant action. Neuropharmacology.

[19] Giachino C, Barz M, Tchorz JS (2014). GABA suppresses neurogenesis in the adult hippocampus through GABAB receptors. Development.

[20] Alvarez-Buylla A, Lim DA (2004). For the long run: maintaining germinal niches in the adult brain. Neuron.

[21] Chesnokova V, Pechnick RN, Wawrowsky K (2016). Chronic peripheral inflammation, hippocampal neurogenesis, and behavior. Brain Behav Immun.

[22] Jiao J, Chen DF (2008). Induction of neurogenesis in nonconventional neurogenic regions of the adult central nervous system by niche astrocyte-produced signals. Stem Cells.

[23] Aimone JB, Li Y, Lee SW (2014). Regulation and function of adult neurogenesis: from genes to cognition. Physiol Rev.

[24] Tajiri S, Oyadomari S, Yano S (2004). Ischemia-induced neuronal cell death is mediated by the endoplasmic reticulum stress pathway involving CHOP. Cell Death Differ.

[25] Jin K, Minami M, Lan JQ (2001). Neurogenesis in dentate subgranular zone and rostral subventricular zone after focal cerebral ischemia in the rat. Proc Natl Acad Sci U S A.

[26] Komitova M, Mattsson B, Johansson BB (2005). Enriched environment increases neural stem/progenitor cell proliferation and neurogenesis in the subventricular zone of stroke-lesioned adult rats. Stroke.

[27] van Praag H, Schinder AF, Christie BR (2002). Functional neurogenesis in the adult hippocampus. Nature.

[28] Reynolds BA, Tetzlaff W, Weiss S (1992). A multipotent EGF-responsive striatal embryonic progenitor cell produces neurons and astrocytes. J Neurosci.

[29] Chojnacki A, Weiss S (2008). Production of neurons, astrocytes and oligodendrocytes from mammalian CNS stem cells. Nat Protoc.

[30] Couillard-Despres S, Winner B, Schaubeck S (2005). Doublecortin expression levels in adult brain reflect neurogenesis. Eur J Neurosci.

[31] McDonald HY, Wojtowicz JM (2005). Dynamics of neurogenesis in the dentate gyrus of adult rats. Neurosci Lett.

[32] Fujioka T, Fujioka A, Duman RS (2004). Activation of cAMP signaling facilitates the morphological maturation of newborn neurons in adult hippocampus. J Neurosci.

[33] Tian L, Nie H, Zhang Y (2014). Recombinant human thioredoxin-1 promotes neurogenesis and facilitates cognitive recovery following cerebral ischemia in mice. Neuropharmacology.

[34] Chen B, Cao H, Chen L (2016). Rifampicin attenuated global cerebral ischemia injury via activating the nuclear factor erythroid 2-related factor pathway. Front Cell Neurosci.

[35] Squire LR (1992). Memory and the hippocampus: a synthesis from findings with rats, monkeys, and humans. Psychol Rev.

[36] Kelly S, McCulloch J, Horsburgh K (2001). Minimal ischaemic neuronal damage and HSP70 expression in MF1 strain mice following bilateral common carotid artery occlusion. Brain Res.

[37] Wu C, Zhan RZ, Qi S (2001). A forebrain ischemic preconditioning model established in C57Black/Crj6 mice. J Neurosci Methods.

[38] Cheng O, Ostrowski RP, Liu W (2010). Activation of liver X receptor reduces global ischemic brain injury by reduction of nuclear factor-kappaB. Neuroscience.

[39] Dave KR, Lange-Asschenfeldt C, Raval AP (2005). Ischemic preconditioning ameliorates excitotoxicity by shifting glutamate/gamma-aminobutyric acid release and biosynthesis. J Neurosci Res.

[40] Liu L, Li C-j, Lu Y (2015). Baclofen mediates neuroprotection on hippocampal CA1 pyramidal cells through the regulation of autophagy under chronic cerebral hypoperfusion. Sci Rep.

[41] Zhu X, Hu H, Li Z (2015). Gua Lou Gui Zhi decoction attenuates post-stroke spasticity via the modulation of GABAB receptors. Mol Med Rep.

[42] Jiang Y, Yang S, Tao J (2016). Opposing needling promotes behavior recovery and exerts neuroprotection via the cAMP/PKA/CREB signal transduction pathway in transient MCAO rats. Mol Med Rep.

[43] Zhu Y-S, Xiong Y-F, Luo F-Q (2019). Dexmedetomidine protects rats from postoperative cognitive dysfunction via regulating the GABA R-mediated cAMP-PKA-CREB signaling pathway. Neuropathology.

[44] Ming G-l, Song H (2005). Adult neurogenesis in the mammalian central nervous system. Annu Rev Neurosci.

[45] Gould E (2007). How widespread is adult neurogenesis in mammals?. Nat Rev Neurosci.

[46] Gonçalves JT, Schafer ST, Gage FH (2016). Adult neurogenesis in the hippocampus: from stem cells to behavior. Cell.

[47] Moon M, Jeong HU, Choi JG (2016). Memory-enhancing effects of Cuscuta japonica Choisy via enhancement of adult hippocampal neurogenesis in mice. Behav Brain Res.

[48] Zang J, Liu Y, Li W (2017). Voluntary exercise increases adult hippocampal neurogenesis by increasing GSK-3β activity in mice. Neuroscience.

[49] Zhang H, Kim Y, Ro EJ (2020). Hippocampal neurogenesis and neural circuit formation in a cuprizone-induced multiple sclerosis mouse model. J Neurosci.

[50] Liu J, Solway K, Messing RO (1998). Increased neurogenesis in the dentate gyrus after transient global ischemia in gerbils. J Neurosci.

[51] Licht T, Kreisel T, Biala Y (2020). Age-dependent remarkable regenerative potential of the dentate gyrus provided by intrinsic stem cells. J Neurosci.

[52] Qiao J, Zhao J, Chang S (2020). MicroRNA-153 improves the neurogenesis of neural stem cells and enhances the cognitive ability of aged mice through the notch signaling pathway. Cell Death Differ.

[53] Lanza M, Fassio A, Gemignani A (1993). CGP 52432: a novel potent and selective GABAB autoreceptor antagonist in rat cerebral cortex. Eur J Pharmacol.

[54] Romei C, Luccini E, Raiteri M (2010). The GABA B receptor antagonists CGP35348 and CGP52432 inhibit glycine exocytosis: study with GABA B1- and GABA B2-deficient mice. Pharmacol Res.

[55] Kleschevnikov AM, Belichenko PV, Faizi M (2012). Deficits in cognition and synaptic plasticity in a mouse model of Down syndrome ameliorated by GABAB receptor antagonists. J Neurosci.

[56] Song J, Zhong C, Bonaguidi MA (2012). Neuronal circuitry mechanism regulating adult quiescent neural stem-cell fate decision. Nature.

[57] Nakagawa S, Kim J-E, Lee R (2002). Regulation of neurogenesis in adult mouse hippocampus by cAMP and the cAMP response element-binding protein. J Neurosci.

[58] Nakagawa S, Kim J-E, Lee R (2002). Localization of phosphorylated cAMP response element-binding protein in immature neurons of adult hippocampus. J Neurosci.

